# Stress Experiences and Support Needs of Nursing Managers in Patient Safety Incidents: A Phenomenological Qualitative Study

**DOI:** 10.1155/jonm/4243800

**Published:** 2026-04-22

**Authors:** Wei Zeng, Yuxin Yang, Li Zhang, Hang Wei, Lijun Jiang, Qingyun Li

**Affiliations:** ^1^ School of Nursing, Nanjing University of Chinese Medicine, Nanjing, China, njucm.edu.cn; ^2^ Department of Nursing, The Second Hospital of Nanjing, Affiliated to Nanjing University of Chinese Medicine, Nanjing, China, njmu.edu.cn

## Abstract

**Background:**

The impact of patient safety incidents on healthcare professionals has drawn increasing attention. As key managerial forces on the clinical frontline, nurse managers not only bear important responsibilities in incident response but may also become “second victims.” The pressure they experience and their need for support warrant systematic investigation.

**Objectives:**

To explore the multidimensional stress experiences and support needs of nurse managers during patient safety incidents, providing a theoretical basis and practical guidance for building scientific management and support systems.

**Design:**

A phenomenological qualitative study.

**Methods:**

Using purposive sampling, 14 nurse managers from a tertiary hospital in Nanjing, China, were recruited for semistructured interviews. Data were analyzed using Colaizzi’s seven‐step method.

**Results:**

Four core themes emerged: (1) complex coping situations under multiple stressors; (2) systemic support deficiencies and dilemmas; (3) growth within the managerial role; and (4) pathways for individual–organizational synergy.

**Conclusion:**

Nurse managers are important yet often overlooked “second victims” in patient safety incidents. Their support needs present distinct institutional and structural characteristics. Healthcare organizations should establish systematic support systems, strengthen psychological follow‐up and collaboration mechanisms, and enhance coping efficacy and organizational resilience among nurse managers.

## 1. Introduction

Patient safety has become a persistent focus of health systems worldwide. The World Health Organization (WHO) defines patient safety as “the avoidance of preventable harm to patients during the process of healthcare,” with the goal of keeping risks within an acceptable range [1]. Patient safety incidents refer to unexpected events occurring during medical care that may result in, or have already caused, physical harm to patients, leading to prolonged hospitalization, disability, or death [2]. According to global statistics [1], patient safety incidents associated with medical safety issues occur an estimated 134 million times annually, resulting in nearly 3 million deaths and economic losses amounting to trillions of US dollars. The vision of the WHO Global Patient Safety Action Plan is that “no one is harmed in healthcare, and every patient receives safe and respectful care” [3].

Although patients are the direct victims of safety incidents, the healthcare professionals involved often experience considerable psychological distress as a result. As early as 1984, Mizrahi et al. [4] began to draw attention to the adverse effects of patient safety incidents on healthcare providers. In 2000, Wu [5] first introduced the concept of the “second victim” in an article, noting that “many errors exist in current procedures and facilities, placing unwitting clinicians and patients in harm’s way. While the patient is the first and most obvious victim of a patient safety incident, the physician is also wounded by the same event: they are the second victim.” In 2009, Scott et al. [6] proposed a widely cited definition of “second victims.” In 2022, the European Research Network on Second Victims (ERNST), drawing on a synthesis of prior evidence, issued a consensus definition: “Any healthcare worker, directly or indirectly involved in an unanticipated adverse patient event, unintentional healthcare error, or patient injury and who becomes victimized in the sense that they are also negatively impacted” [7].

The second victim phenomenon (SVP) is prevalent worldwide. Studies have shown [8] that between 10% and 86% of healthcare professionals experience a patient safety incident during their career and are affected by it, with approximately half reporting at least one episode of being a second victim [9]. A study of nurses in different workplace settings in Germany found that 60% had experienced SVP [10]. Similarly, a cross‐sectional survey in mainland China reported that 45.26% of nurses had encountered at least one patient safety incident [11]. Such events not only affect the mental health of healthcare professionals but may also trigger physical or behavioral responses such as insomnia, loss of appetite, and professional burnout [12, 13].

With “caring for nurses” increasingly recognized as a strategic priority in global nursing development, the need to protect nurses as “second victims” has gained growing attention. The International Council of Nurses (ICN) has designated the theme of International Nurses Day 2025 as “Our Nurses. Our Future. Caring for nurses strengthens economies,” once again underscoring the critical importance of nurses’ health for the sustainable development of health systems and the stability of social and economic structures [14]. In 2021, the General Office of the State Council of China issued the Opinions on Promoting the High‐Quality Development of Public Hospitals, which emphasized strengthening quality construction in public hospitals, prioritizing the protection, care, and support of healthcare workers, safeguarding their legitimate rights and interests, and establishing long‐term mechanisms to enhance occupational safety and job satisfaction [15].

In recent years, research on frontline nurses as second victims has increased, with scholars conducting in‐depth explorations from perspectives such as stress responses, professional burnout, and organizational support [16–18]. In modern healthcare systems that prioritize patient safety, nurse managers, as frontline clinical leaders, play critical roles in coordinating resources, controlling risks, and maintaining team stability. When a patient safety incident occurs, they must not only rapidly organize emergency response and incident investigation but also manage multiple tasks such as consoling families and supporting nurses, making them a central force in incident management [19, 20]. First‐line nurse managers commonly experience role conflict, high job demands, and limited organizational support, all of which may further compound the psychological impact of patient safety incidents and increase their risk of becoming second victims [19, 21]. However, existing studies on second victims during patient safety incidents have largely focused on frontline clinical nurses, with insufficient attention paid to the stress, challenges, and support needs of nurse managers.

To gain an in‐depth understanding of nurse managers’ psychological experiences and support needs during patient safety incidents, this study adopted the Stress‐Coping Theory [22] and Organizational Support Theory [23] as its theoretical framework. Proposed by Lazarus and Folkman [22], the Stress‐Coping Theory emphasizes that when individuals face a stressful event, they first conduct a primary appraisal (assessing whether the event poses a threat), followed by a secondary appraisal (evaluating whether they have the resources to cope with it), and then select either problem‐focused or emotion‐focused coping strategies accordingly. This theory provides valuable insights into nurse managers’ cognitive appraisals, psychological reactions, and strategy selection during such incidents. Organizational Support Theory centers on individuals’ perceptions of whether their organization values their contributions and cares about their well‐being. Evidence indicates that higher perceived organizational support is associated with stronger emotional recovery and lower risk of professional burnout in stressful situations [23]. For nurse managers, the level of organizational support directly influences their psychological recovery and coping effectiveness when facing complex incidents.

Therefore, this study focuses on nurse managers’ experiences during patient safety incidents, guided by the Stress‐Coping Theory and Organizational Support Theory. Using a phenomenological qualitative design, it explores the multidimensional experiences and needs of nurse managers in incident response, providing both theoretical and practical implications for improving organization‐level coordinated support mechanisms.

## 2. Methods

### 2.1. Design

This study adopted a phenomenological qualitative design. Phenomenology emphasizes an in‐depth understanding of individuals’ “lived experiences,” with particular attention to subjective feelings and meaning‐making within specific contexts [24]. Given that the aim of this study was to explore the multidimensional experiences and needs of nurse managers during patient safety incidents, this approach aligned with the philosophical foundation of phenomenology, which seeks to reveal the essence of experiences from the individual’s perspective within real‐life events. The study was reported in accordance with the Consolidated Criteria for Reporting Qualitative Research (COREQ) checklist [25].

### 2.2. Participants

This study was conducted in a tertiary hospital in Nanjing, China. Purposive sampling combined with a maximum variation strategy was employed to obtain rich and representative experiential data from nurse managers. The research team predefined variation dimensions prior to recruitment, including clinical unit type (e.g., medical, surgical, and intensive/critical care units), professional rank, years in a managerial role at the time of the incident, and incident type (e.g., medication/administration‐related errors, tube/line dislodgement, falls, and suicide). Inclusion criteria were (1) at least 1 year of experience in clinical nursing management; (2) having been involved in the management of a patient safety incident in their department; (3) good communication skills and the ability to articulate personal feelings clearly; and (4) providing informed consent and willingness to participate in the study. With support from the nursing department, the research team compiled a list of eligible candidates. Recruitment proceeded iteratively, with purposive invitations to ensure coverage of underrepresented units/dimensions. All participants were female, which reflects the gender composition of nursing management positions at the study site. Using a concurrent data collection and analysis approach, the sample size was determined by information saturation [26]. Saturation was assessed iteratively by two independent coders through ongoing coding after each interview and was operationally defined as the point at which two consecutive interviews generated no new codes/meaning units and the thematic structure remained stable. A total of 14 nurse managers were interviewed; preliminary saturation was achieved after the 12th interview, with two additional participants recruited to confirm saturation. To protect participants’ privacy, identifiers R1–R14 were used in place of real names.

### 2.3. Research Team Characteristics

The research team consisted of six members, including one male and five females: four nursing postgraduate students (ZW, YYX, JLJ, and WH), one nurse manager (ZL), and one nursing research scholar (LQY). All four postgraduate students had received systematic training in qualitative research methods and possessed strong interviewing and analytical skills; among them, ZW and YYX were responsible for conducting the interviews in this study. ZL, a nurse administrator from the nursing department of a tertiary hospital in Nanjing, China, had over 30 years of clinical nursing and management experience, with extensive involvement in organizing and managing patient safety incident responses. She also served as a postgraduate supervisor in nursing and had a deep understanding of the study topic. LQY had long been engaged in nursing research, with substantial experience in qualitative methodologies, and provided methodological guidance for this study.

### 2.4. Data Collection

Data were collected through semistructured, face‐to‐face interviews. Based on a literature review and guided by the Stress‐Coping Theory [22] and Organizational Support Theory [23], a preliminary interview guide was developed. This guide was reviewed by several experts with extensive experience in qualitative research. Prior to the formal interviews, two pilot interviews were conducted with nurse managers who met the inclusion criteria. Feedback from these pilots was used to refine the language and structure of the guide (see Table 1). Interviews were conducted in a quiet, private consultation room or teaching room within the hospital. Appointments were arranged in advance to ensure a calm and undisturbed environment. Before the interviews, the researchers provided participants with detailed information about the study’s purpose, the core concept of the “second victim,” and the interview process, while building rapport with them. All interviews were audio‐recorded, and nonverbal expressions—such as facial expressions, emotional fluctuations, and body movements—were documented. When participants’ responses were unclear or could be further elaborated, the interviewer used techniques such as paraphrasing key points and appropriate probing to accurately capture participants’ authentic thoughts and perspectives. Only one participant declined audio‐recording. The interviewer, therefore, used real‐time written note‐taking to document the interview in full and expanded and refined the notes into a complete textual record within 24 h after the interview, while also paraphrasing key points for immediate on‐site confirmation. Each interview lasted 20–30 min. All participants took part voluntarily, signed informed consent forms, and none withdrew or declined participation. Data collection took place between 22 June and 06 August 2025 at a tertiary hospital in Nanjing, China. No repeat interviews were conducted during the study period.

**TABLE 1:** Interview guide.

Question
1. Please describe the most memorable patient safety incident in your managerial career and provide detailed information about the circumstances at the time
2. During the process of handling this incident, what kinds of stress did you experience? How did these stresses affect you?
3. In your opinion, what was the most difficult problem to address during the management of this incident? What kinds of support did you most hope to receive?
4. When you noticed that a nurse became self‐blaming, anxious, or emotionally low because of the incident, what measures did you take to help her? How effective were these measures?
5. After experiencing this incident (or handling multiple similar patient safety incidents), did you have any reflections or insights?
6. In your view, what shortcomings or areas for improvement exist in the current hospital process for managing patient safety incidents? What specific suggestions do you have?

### 2.5. Data Analysis

Within 24 h after each interview, the first author (ZW) transcribed the audio recordings verbatim using Word 2021. Another researcher (YYX) then reviewed the original recordings while checking the transcripts to ensure completeness and accuracy of the data. Data collection and analysis were conducted concurrently. Colaizzi’s seven‐step method [27] was employed for data analysis, with NVivo 14 software used to facilitate coding and data management. Notably, the expanded note‐based text was included in the coding and analysis alongside the audio‐recorded transcripts; however, we avoided using verbatim quotations from this participant in the results and ensured that all themes were supported by multiple audio‐recorded transcripts. The specific analytical steps were as follows: (1) The researchers repeatedly read each interview transcript to gain an in‐depth understanding of the situations, emotions, and feelings experienced by participants during patient safety incidents; (2) two researchers independently extracted, paragraph by paragraph and sentence by sentence, significant statements directly relevant to the study aims (e.g., sources of stress, emotional/physiological responses, coping behaviors, organizational support, and support needs); (3) the researchers condensed these significant statements to develop meaning units that remained close to the original intent (prioritizing participants’ own words or near‐synonymous expressions) and then returned to the full transcript context to verify that the meaning units did not deviate from participants’ original meanings; (4) meaning units with similar content or underlying meanings were grouped together to form preliminary themes, such as “sources of stress,” “emotional reactions,” “physiological reactions,” and “lack of organizational support;” (5) building on these preliminary themes, the researchers expanded and integrated the content in conjunction with the original data to produce a comprehensive description of nurse managers’ experiences during patient safety incidents, for example, “severity and uncertainty of the incident” and “poor collaboration within the healthcare system;” (6) through further abstraction and comparative analysis, core themes reflecting the essence of the phenomenon were extracted, revealing the key psychological processes and support needs of nurse managers in patient safety incidents, such as “complex coping scenarios under multiple stressors” and “growth within the managerial role;” and (7) three participants were randomly selected for face‐to‐face follow‐up interviews. They were provided with an anonymized theme/subtheme framework and brief descriptions of each theme (with representative quotations) and were asked to verify whether the themes reflected their experiences and whether any omissions or misunderstandings were present. The feedback was consistent and did not result in any substantive revisions.

### 2.6. Rigor

To ensure the trustworthiness of this study and minimize research bias, rigorous quality control measures were implemented throughout the stages of study design, data collection, and analysis.

During sample selection, purposive sampling combined with the principle of maximum variation was used to enhance both the breadth and depth of the data. To reduce researcher bias, the investigators maintained a “bracketing” stance during interviews and analysis, respecting participants’ original meanings and avoiding personal subjective judgments that might influence the analytic process. Methodological triangulation was applied extensively. In the data collection phase, both interviews and observations were used to provide a comprehensive perspective on the phenomenon. In the data analysis phase, two researchers (ZW and YYX) independently conducted the extraction of significant statements, construction of meaning units, and thematic development. Any discrepancies were first resolved through item‐by‐item discussion with reference to the original transcript context; if consensus could not be reached, a third researcher reviewed the relevant materials and provided recommendations. Final agreement was achieved through team discussion, ensuring the accuracy of theme identification. To enhance credibility, member checking was conducted with three randomly selected participants to verify that the analysis results were consistent with their actual experiences. Complete audit trails were maintained throughout the research process, including documentation of interview guide development, verbatim transcripts, coding procedures, and the pathways to theme formation. Original participant quotations were included in the presentation of findings to strengthen the verifiability and transparency of the results.

### 2.7. Ethical Considerations

This study was approved by the Ethics Review Committee of Nanjing Hospital, Affiliated to Nanjing University of Chinese Medicine (approval no. 2025‐LS‐ky65). All participants voluntarily signed informed consent forms after being fully informed of the study’s purpose, content, and potential risks. During the research process, participants retained the right to ask questions and withdraw from the study at any time without any consequences. To protect participants’ privacy and data security, anonymization and deidentification procedures were applied throughout the study. Participants were assigned codes (R1–R14) instead of their real names, and any potentially identifying personal or unit‐related information was removed or replaced during transcription and data management. Audio recordings, transcripts, and all related materials were stored in encrypted files and were accessible only to authorized members of the research team; all data were centrally managed by the first author and could not be copied, shared externally, or used for purposes beyond this study without permission. When reporting the findings, only anonymized quotations were presented and any details that might enable participant identification were omitted, thereby maximizing protection of participants’ privacy and rights.

## 3. Results

A total of 14 nurse managers were interviewed in this study. Participant characteristics are presented in Table 2. Four core themes and 13 subthemes were identified (Table 3). The four core themes were (1) complex coping situations under multiple stressors; (2) systemic support deficiencies and dilemmas; (3) growth within the managerial role; and (4) pathways for individual–organizational synergy.

**TABLE 2:** Participant characteristics (*n* = 14).

Number	Gender	Age (years)	Marital status	Education	Professional title	Department	Years in current specialty management at time of incident	Type of incident
R1	Female	38	Single	Bachelor’s degree	Senior	Gynecology	3	Wrong medication sequence
R2	Female	39	Married	Bachelor’s degree	Senior	Cardiothoracic Surgery	5	Patient misidentification
R3	Female	39	Married	Bachelor’s degree	Intermediate	Respiratory and Critical Care Medicine	2	Patient misidentification
R4	Female	37	Married	Master’s degree	Senior	Hepatobiliary and Pancreatic Surgery	5	Tube dislodgement
R5	Female	39	Married	Bachelor’s degree	Senior	Gastrointestinal Treatment Center	3	Medication error
R6	Female	39	Married	Bachelor’s degree	Senior	ICU	7	Pressure injury
R7	Female	37	Married	Master’s degree	Intermediate	Obstetrics and Gynecology	1	Patient misidentification
R8	Female	39	Married	Bachelor’s degree	Senior	Oncology	4	Incorrect infusion rate
R9	Female	38	Married	Master’s degree	Intermediate	Geriatrics	1	Accidental fall
R10	Female	40	Married	Master’s degree	Senior	Infectious Diseases	2	Infusion extravasation
R11	Female	49	Married	Bachelor’s degree	Senior	General Surgery	29	Patient suicide
R12	Female	55	Married	Bachelor’s degree	Senior	Endoscopy Center	4	Missed colonoscopy
R13	Female	38	Married	Master’s degree	Senior	ICU	3	Respiratory tube dislodgement
R14	Female	39	Married	Master’s degree	Senior	Gastroenterology	6	Accidental fall

**TABLE 3:** Summary of themes.

Core theme	Subtheme	Meaning unit
Complex coping situations under multiple stressors	Severity and uncertainty of the incident	Actual or potential harm to patients
		Frequent occurrence or serious nature of incidents
	Managerial role stress and overlapping conflicts	Overlapping managerial tasks; cross‐departmental coordination burden
		Nurse–patient communication conflicts; external blame pressure
	Persistent psychological and physiological stress reactions	Negative emotional experiences
		Immune dysregulation; endocrine disorders
		Sleep disturbances; distressing dreams
	Impaired work performance and professional beliefs	Decreased work efficiency
		Undermined professional confidence

Systemic support deficiencies and dilemmas	Poor internal collaboration within the healthcare system	Ineffective nurse–physician collaboration
		Insufficient hospital system support
	Limited support measures with minimal effectiveness	

Growth within the managerial role	Strengthened incident‐handling capabilities	Maintaining a calm mindset
		Timely actions to prevent escalation
	Enhanced managerial competence	Improved theoretical knowledge to enhance decision‐making
		Tailoring strategies to individuals
	Role transition and reconstruction of professional identity	Transition from “passive responder” to “proactive manager”
		Reconstruction of professional identity

Pathways for individual–organizational synergy	Optimizing psychological support mechanisms	Reduction of public criticism
		Positive encouragement and support
		Psychological assessment and follow‐up
	Clarifying responsibility mechanisms	Strengthening responsibility among junior nurses
	Organizational‐level guidance and collaboration	Institutionalization of medical collaboration
		Clear procedures for incident reporting and handling
	Establishing graded, categorized, and closed‐loop handling mechanisms	Stratified handling based on incident severity
		Patient condition tracking and follow‐up

### 3.1. Theme 1: Complex Coping Situations Under Multiple Stressors

#### 3.1.1. Subtheme 1.1: Severity and Uncertainty of the Incident

In most participants’ accounts, the primary source of stress from patient safety incidents stemmed from the severity of the event itself. This was particularly evident when incidents involved high‐alert medications or special patient groups, such as pregnant women, infants, and elderly patients. In such situations, nurse managers commonly experienced intense unease and persistent mental tension.

“It might be something quite serious… if the patient went into shock… just thinking about it makes me feel terrified.” (R5)

“Because we are in obstetrics and gynecology, it involves two lives—an adult and a child… when I heard it, my mind just went completely blank.” (R7)

Beyond the immediate impact of the incident, what caused even greater stress for nurse managers was the uncertainty of its subsequent development. Participants noted that even after handling the incident, they continued to monitor the patient’s condition, the family’s attitude, and the potential risk of disputes. This process of “waiting to see whether potential risks would materialize” was often described as more anxiety‐provoking than the incident itself.

“Because the patient was a child, only one year old, and there was a large amount of extravasated medication… I had no idea what the outcome would be.” (R10)

Some participants further mentioned that if the incident involved a fundamental error—such as failure to perform the “three checks and seven rights” or to conduct ward rounds within the required timeframe—once harm occurred, it would bring even greater institutional pressure. Such pressure was particularly pronounced when similar incidents occurred repeatedly within a short period, which often led to feelings of frustration and helplessness.

“Two cases happened in the same month… it was really overwhelming.” (R8)

“This incident was a complete violation of the core system (frowns)… after all, there was a nursing fault on our side, which directly led to this event.” (R12)

#### 3.1.2. Subtheme 1.2: Managerial Role Stress and Overlapping Conflicts

In managing patient safety incidents, nurse managers often had to simultaneously undertake multiple responsibilities, including incident investigation, staff reassurance, patient condition follow‐up, and cross‐departmental coordination. Participants described themselves as “the hub” connecting various systems, with overlapping duties that often left them mentally and physically exhausted.

“First, I need to understand the cause and effect of the incident; second, I have to protect the nurse; and third, I must try to avoid any disputes or conflicts… (sighs) all the pressure just ends up on my shoulders.” (R14)

When incidents involved multiple departments, nurse managers had to mediate between different positions and interests to facilitate problem resolution, while also maintaining good interpersonal relationships to prevent escalation of interdepartmental conflicts.

“Such coordination inevitably brings people from different departments together. Some departments may not want the incident to be made open and transparent… some other department leaders still hold certain opinions about you (sighs).” (R1)

Questioning from both patients’ families and within the hospital added further pressure. Several participants mentioned feeling powerless when facing the anger and accusations from patients’ families, while disapproval and strict attitudes from the medical side further intensified their psychological burden.

“After the incident happened, at first only one person from the patient’s side was here accompanying them, but later they called in many others… it felt really aggressive.” (R3)

“Our department director has extremely high expectations… they immediately denied us, saying it was entirely nursing’s fault, and made me face the patient and family alone.” (R8)

#### 3.1.3. Subtheme 1.3: Persistent Psychological and Physiological Stress Reactions

Most participants, when recalling patient safety incidents, reported experiencing intense and complex negative emotions, commonly manifested as fear, anxiety, loss, and self‐blame. The intensity of these psychological reactions was often closely related to the severity of the incident, the attitude of the patient’s family, or conflicts within the organization.

“…I was on the verge of breaking down, really terrified.” (R7)

“After the incident happened, anxious, anxious—just extremely anxious.” (R8)

These stress reactions were not limited to the psychological level. Some participants mentioned that the incident triggered significant physical health problems, such as immune system disorders, recurrent allergies, or endocrine imbalances. Sleep disturbances were also frequently reported. Several participants noted that during the incident management phase and for a considerable period thereafter, they experienced difficulty falling asleep or were troubled by recurring dreams, often with content directly related to the incident.

“Because of this incident, I developed an allergy a week later… my thyroid function has still not returned to normal.” (R11)

“I dreamed about the patient having a miscarriage… and then something unexpected happening again.” (R1)

#### 3.1.4. Subtheme 1.4: Impaired Work Performance and Professional Beliefs

Some nurse managers continued to experience negative effects for a considerable period after the occurrence of a patient safety incident. Participants reported that during incident handling, they were required to attend multiple meetings, prepare written reports, and respond to inquiries, which disrupted their usual work rhythm, made it difficult to concentrate, and reduced work efficiency. Some even developed avoidance tendencies toward the patient involved.

“Before the patient was discharged, I felt a constant weight in my heart—about a week—and I was somewhat afraid to face the patient.” (R1)

“It had quite a significant impact on my work status… because I had to keep attending meetings and conducting warning education.” (R12)

In some cases, the incidents further prompted nurse managers to question their own professional competence. Several participants stated that despite making every effort to correct problems and respond effectively during incident management, they were still unable to prevent the occurrence or escalation of the event. This led to self‐doubt regarding their professional abilities, and in some instances, even thoughts of resignation.

“…Am I not competent enough… my position was even reassigned (bitter laugh), and for many reasons, I felt that the department director had denied me (tears). At that time, I actually thought about quitting—resigning.” (R8)

### 3.2. Theme 2: Systemic Support Deficiencies and Dilemmas

#### 3.2.1. Subtheme 2.1: Poor Internal Collaboration Within the Healthcare System

In handling patient safety incidents, some participants reported that good nurse–physician collaboration within their department helped share the workload and improve incident management efficiency. However, more participants described obstacles in collaborating with the medical side, noting that physicians often had a low level of involvement in incident response. In many cases, especially when communicating with patients and their families, nurses bore sole responsibility for explanation and reassurance. This unbalanced division of responsibilities further increased the psychological pressure and coping burden for nurse managers. Some participants further pointed out that physicians possess greater professional authority and are more likely to gain the trust of patients and their families, which can help ease tension and conflict during communication. In practice, however, it was often the nurse alone who faced the patient side, without the collaboration and support of a multidisciplinary team.

“If communication between doctors and nurses is poor, then during incident handling you feel like you’re left hanging in midair.” (R1)

“I really hope a doctor could join us in comforting the patient—they have authority. For example, oncologists or pharmacists… they may be more convincing.” (R2)

In addition to the lack of medical collaboration, participants also emphasized insufficient support at the hospital system level, which further complicated incident management. Whether in terms of coordination mechanisms, information systems, or the adequacy of physical infrastructure, the available support was perceived as inadequate.

“We are a geriatrics department, but our hospital hasn’t implemented any age‐friendly modifications.” (R9)

“…A lot of issues could actually be prevented through digital systems, but right now we have to rely on manual intervention.” (R5)

#### 3.2.2. Subtheme 2.2: Limited Support Measures With Minimal Effectiveness

Some participants noted that after a patient safety incident, certain supportive actions were taken for the nurses involved, such as providing emotional comfort or arranging rest. However, the effectiveness of these measures was perceived as limited. Most current support efforts remain at the managerial or humanistic care level, lacking professional and systematic psychological support capable of providing in‐depth and lasting intervention for the psychological or physiological impact on the nurses involved. In some cases, although the nurses appeared to have returned to work, their negative emotional states had not been fully alleviated and continued to persist.

“…They were very remorseful and emotionally low, probably still thinking about why they made that mistake at the time.” (R3)

“When that nurse was involved in the incident, they were also very nervous and scared… it lasted for a while, but I don’t know exactly how long.” (R8)

### 3.3. Theme 3: Growth Within the Managerial Role

#### 3.3.1. Subtheme 3.1: Strengthened Incident‐Handling Capabilities

Experiencing patient safety incidents gradually made nurse managers more aware of the importance of maintaining a calm mindset in unexpected situations. Participants noted that emotional fluctuations are inevitable when facing sudden events, but with accumulated experience, they learned how to control their emotions and maintain rational judgment under external pressure. Even when feeling tense and uneasy inside, they recognized the need to remain outwardly composed.

“Mindset is very important (nods). You must keep calm and composed—never panic. Even if you panic inside, you can’t show it on the outside.” (R14)

“When dealing with problems, you must stay rational… you can’t panic or be at a loss.” (R11)

Alongside this calm mindset came a deeper appreciation for the importance of timeliness. Many participants emphasized that in the very first moments after an incident, rapid response and timely intervention are key to preventing escalation—such as knowing which physician to call for the fastest assistance or how to activate emergency protocols. They stressed that only by managing the pace in the initial handling stage can the subsequent development of the event be effectively controlled.

“Once a patient safety incident occurs… minimizing harm is critical… you need to know which doctor to call to get the fastest result.” (R7)

“If there’s a heated verbal dispute or some behavioral conflict, you must put the patient and family in a relatively quiet room with fewer people to avoid disturbing other patients’ treatment.” (R14)

#### 3.3.2. Subtheme 3.2: Enhanced Management Competence

Through repeated practice in handling patient safety incidents, some nurse managers gradually shifted from experience‐based responses to more strategic governance. They recognized that relying solely on experience and intuition was insufficient for effectively addressing the complexity and variability of the clinical environment. Instead, they needed to draw on theoretical tools and systematic processes to support problem identification, risk assessment, and team collaboration.

“We used root cause analysis… when you apply such tools in management… your thinking becomes much clearer… using theory to guide practice.” (R8)

“The fishbone diagram is really useful. Analysing a problem from the perspectives of people, equipment, materials, methods, and environment always helps us identify the cause of the incident.” (R6)

With accumulated experience, some participants reported that in their daily work, they began moving away from a “one‐size‐fits‐all” management approach. They increasingly considered the unique personality traits, emotional states, and career stages of the individuals involved, handling incidents based on these differences to avoid adding new pressures through oversimplified measures.

“Assess the person’s character traits and work status, analyze the situation case by case, and adopt different communication methods…” (R12)

#### 3.3.3. Subtheme 3.3: Role Transition and Reconstruction of Professional Identity

Through repeated experiences in handling patient safety incidents, nurse managers gradually shifted from being “passive responders” to “proactive managers.” They moved from merely bearing responsibility and making remedial efforts after the fact to engaging in systematic reflection and preventive management, identifying the deeper root causes of incidents. Some managers incorporated typical cases into routine training programs, using real‐world examples to enhance nurses’ sensitivity to patient safety management and their sense of responsibility, thereby continuously strengthening the team’s risk awareness.

“So this case… we study it repeatedly, use it as a warning…” (R13)

“…Hold meetings to discuss, find the cause, and examine exactly which step went wrong—making sure that the same thing never happens again.” (R12)

As their management experience accumulated and their handling skills improved, nurse managers’ sense of identification with their professional role also grew stronger. Although the position carries significant responsibility and pressure, the ability to maintain order in complex situations and guide the team in resolving risks gave them a sense of professional value and accomplishment.

“I think in this management position, you must have inner strength… as long as the patient is fine, that’s what matters.” (R8)

“…At least I feel that this incident didn’t affect a nurse’s work or life—that’s what makes me most gratified.” (R11)

### 3.4. Theme 4: Pathways for Individual–Organizational Synergy

#### 3.4.1. Subtheme 4.1: Optimizing Psychological Support Mechanisms

In the interviews, nurse managers generally indicated that current psychological support systems for personnel involved in patient safety incidents remain inadequate. Some participants pointed out that certain organizational practices may actually increase the psychological burden on the nurses involved. In particular, public “self‐criticism”–style case sharing was perceived as a form of “secondary harm,” intensifying feelings of shame and guilt and potentially delaying psychological recovery. To reduce the burden on the individuals involved, some departments attempted to have the nurse manager report on their behalf. However, as the frequency of incidents increased, nurse managers themselves also bore significant emotional strain from repeated organizational communication and reporting, making their own psychological well‐being a matter of concern.

“Actually, from the start I felt that having the person involved read it aloud at the head nurse meeting is really a secondary harm…” (R7)

“…When we have to report it, the pressure is no less; after going up there many times, it feels pretty embarrassing… it’s a big pressure on the manager.” (R12)

Some participants emphasized the importance of positive reinforcement and emotional support. They believed that in handling patient safety incidents, the organization should not only focus on determining responsibility but also recognize the managers’ postincident response efforts and coordination abilities, thereby enhancing their sense of professional value and motivation to cope.

“…There needs to be more care for head nurses, and recognition and praise for the good aspects of our work.” (R8)

“I hope leaders can provide more support for our work and give us the motivation to keep going.” (R5)

In addition, some participants suggested that psychological support should evolve from ad hoc consolation to a systematic and institutionalized process of assessment and follow‐up. They recommended establishing a psychological evaluation system for both nurses and managers involved in incidents, tracking stress responses at different stages after the event, and providing targeted psychological interventions and resource support.

“That is to say, carry out a psychological counseling and debriefing session for all participants… I think that’s necessary.” (R11)

“For the nurse involved, follow‐up could include a psychological assessment at 12, 24 h after the incident.” (R10)

#### 3.4.2. Subtheme 4.2: Clarifying Responsibility Mechanisms

The current responsibility‐sharing system within nursing teams remains underdeveloped. In particular, during accountability and reflection processes following patient safety incidents, junior nurses often occupy a “low‐responsibility, low‐involvement” peripheral role. Some participants pointed out that when incidents are caused by rotating nurses or junior staff, the responsibility is often assumed by the preceptor or the nurse manager, leaving the individuals involved without direct pressure to face the consequences or opportunities for deep reflection. As a result, their sense of professional responsibility is difficult to cultivate. It was suggested that a more reasonable responsibility mechanism be established according to nurses’ seniority, job responsibilities, and work attitudes, with clear delineation of accountability in incidents so that standardized practices and a sense of responsibility are reinforced in daily work.

“Trainees and rotating nurses lack performance constraints… they feel that if an incident happens, it just happens… all the pressure is on the preceptor and the head nurse, and I can’t penalize them…” (R2)

#### 3.4.3. Subtheme 4.3: Organizational‐Level Guidance and Collaboration

Several nurse managers noted that the effective handling of patient safety incidents depends not only on individual coping abilities and team responses but also on robust interdepartmental coordination mechanisms and institutional support at the organizational level. Some participants suggested formalizing departmental collaboration, with clearly defined roles and communication procedures for each department in the event of a patient safety incident. Institutional safeguards would ensure smoother coordination and stronger collaborative capacity.

“When an incident occurs, the medical and nursing staff should face it together… at that point, everyone should avoid blaming each other… it might make handling the situation easier.” (R10)

“There should be an institutional policy requiring doctors and nurses to manage incidents collaboratively… it shouldn’t depend on individual initiative.” (R8)

Participants also pointed out that while incident reporting systems exist, in practice, certain stages often involve repeated revisions. They recommended further refining and standardizing incident reporting and handling procedures to improve clarity and reduce management stress caused by ambiguous processes.

“If, when writing the incident report, I were clearly told what to include, I probably wouldn’t need to revise it over and over again.” (R11)

#### 3.4.4. Subtheme 4.4: Establishing Graded, Categorized, and Closed‐Loop Handling Mechanisms

In the practical management of patient safety incidents, a graded and categorized response mechanism should be established. Some current “one‐size‐fits‐all” approaches fail to reflect the heterogeneity of incidents, which not only reduces efficiency but may also impose unnecessary psychological burdens on the personnel involved. Participants suggested adopting differentiated handling strategies according to the severity of the incident and its impact on the patient.

“I think some incidents really only need to be discussed within the department. But if it’s particularly serious… causing very severe harm to the patient… then of course it has to be reported… it should also serve as a warning to other departments.” (R9)

“After an incident, we need to deal with it case by case. I think the approach should vary depending on the nature of the incident.” (R6)

Participants also proposed incorporating patient safety incident follow‐up into a closed‐loop management process. In particular, for patients with confirmed harm, regular follow‐up after discharge to monitor changes in their condition in a timely manner was considered not only a clinical necessity but also a means of providing psychological comfort and rebuilding trust.

“If the incident caused significant harm to the patient… it’s necessary to track and follow up on the patient’s condition, which can also provide them with some reassurance.” (R10)

## 4. Discussion

This study delineates nurse managers’ sources of stress, stress responses, gaps in organizational support and support needs, as well as their role development and recommendations for system improvement in the management of patient safety incidents, thereby adding to the evidence based on the SVP. In contrast to prior research that has predominantly focused on frontline nurses, our study shifts attention to unit‐level nurse managers and illustrates the multiple constraints they face during incident management and the types of support they consider most salient. These findings broaden and refine current understanding of second‐victim experiences within the nursing workforce.

Theme 1 shows that nurse managers experience complex, multisource and multilayered pressures when responding to patient safety incidents. Stress arises not only from the abrupt and uncertain nature of incidents (e.g., patient harm and the potential for disputes) but also from the breadth of managerial responsibilities they must assume, including incident investigation, supporting and reassuring staff, coordinating across departments, and reporting to senior leadership [20, 28]. As a result, nurse managers often become the point at which multiple stressors converge—an intersection of responsibility and strain that distinguishes them from frontline nurses. We further found that incident severity and patients’/families’ reactions had substantial psychological impacts on nurse managers; some participants also reported physical manifestations such as sleep disturbance and immune‐related problems. This is consistent with Li et al. [29], who identified incident severity and patient/family attitudes as key triggers of trauma‐related psychological responses among nursing staff. Mintzberg’s managerial role theory [30] suggests that managers are required to perform multiple role functions simultaneously in their work. Following a patient safety incident, nurse managers face not a single source of role‐related stress but rather the compounded challenge of multiple overlapping role conflicts: they must participate in incident review while also supporting the nurses involved and communicating with patients’ families; they must coordinate across departments while at the same time responding to questioning and criticism from medical staff and senior leadership. This dual task structure of “horizontal coordination and vertical pressure” can easily lead to psychological and physiological exhaustion among nurse managers and may even increase their intention to leave their positions [28, 31, 32]. Taken together, the observed cascade of “emotional exhaustion–self‐doubt–burnout” suggests that second‐victim distress among nurse managers is not only present but may be more concealed and less likely to be recognized promptly than that experienced by frontline nurses.

Nurse managers operate in complex situations characterized by overlapping pressures during patient safety incidents, facing challenges that extend beyond professional responsibilities to encompass psychological, physiological, and professional identity dimensions. This phenomenon highlights the need for healthcare institutions to recognize frontline management staff as “invisible victims” and to establish mechanisms for stress monitoring and psychological support. Such measures can help prevent nurse managers from falling into a “coping while being harmed” cycle, thereby safeguarding their competence and effectiveness in high‐pressure environments.

Theme 2 highlights that, in managing patient safety incidents, nurse managers frequently confront system‐level gaps and barriers to interdepartmental collaboration. These shortcomings can amplify their stress burden and undermine the overall effectiveness of incident response. Following incidents, nursing staff often reported a lack of substantive medical collaboration and support, which weakened the professionalism and authority of communication—an issue also noted in prior studies [33]. The quality of physician–nurse collaboration is central to building a strong patient safety culture; positive collaboration not only improves clinical communication and reduces errors but also alleviates workload and psychological strain among team members [34]. In contrast, inadequate interprofessional collaboration erodes the support system for frontline nurses and leaves nurse managers with limited resources when dealing with complex events, increasing the risk of emotional exhaustion [35]. Beyond gaps in interprofessional support, participants described broader organizational deficiencies that further constrained incident management. Outdated information systems and inadequate infrastructure meant that risk alerts—many of which could be automated—still depended on manual processes, increasing the likelihood of human error and adding to managerial workload. Prior research has emphasized that effective interdisciplinary teamwork is a key driver of healthcare quality and patient safety [36, 37]. Importantly, patient safety incidents typically reflect the accumulation of multiple system vulnerabilities rather than isolated individual mistakes. When confronted with such structural risks in the absence of institutional support, nurse managers may be pushed into the unsustainable position of compensating for system shortcomings through additional human effort. Our findings also indicate that existing support for frontline nurses is often limited in scope and of limited effectiveness (e.g., brief reassurance without professional, structured interventions). Such support is frequently informal and noninstitutionalized, lacking continuity and specialist input, and may, therefore, fail to address the deeper psychological needs of those involved—consistent with Wang et al. [38]. Crucially, these “surface‐level” approaches may not meaningfully reduce trauma‐related distress among frontline nurses; instead, they can shift the burden of emotional soothing and communication back onto nurse managers. Studies have shown that nurse managers play a crucial role in supporting nurses as second victims and coordinating additional support resources, while nurses also generally expect timely and effective support from nurse managers following patient safety incidents [39, 40]. However, when organizational support is not institutionalized, nurse managers often become not only providers of support but also secondary bearers of stress, further increasing their burden.

Patient safety incidents expose not only vulnerabilities in clinical practice but also structural deficiencies in organizational support. The pressures borne by nurse managers arise not merely from the incidents themselves but from a broader “structural burden” created by weak collaboration mechanisms, lagging resource systems, and inadequate institutional responses. Going forward, strengthening interprofessional collaboration, upgrading information and infrastructure support, and building a multilevel, systematic support network will be essential to improving the quality of incident response while safeguarding nurse managers’ psychological wellbeing and professional safety.

Although patient safety incidents are inherently stressful and complex, our findings suggest that they can also serve as catalysts for role development among nurse managers. Theme 3 (“Growth in the managerial role”) indicates that, through managing patient safety incidents, nurse managers reported multidimensional progress in coping capacity, management strategies, and role identity. This growth reflects more than adaptation to external demands; it also represents an internalized shift from experience‐based responses to strategic approaches and from emotion regulation to professional governance. Participants described learning to regulate their emotions, remain composed, and make rapid judgments in unpredictable, high‐stakes situations. This capacity was not framed as a fixed personal trait but as resilience and professional intuition cultivated through repeated incident management experiences. Zhang et al. [19] referred to this process as “forced growth,” whereby repeated exposure to incident handling gradually builds psychological defenses and crisis‐management capability. Importantly, participants’ accounts suggested that this development was not purely passive: as they gained greater proactivity and discernment over time, it evolved into a more deliberate internal transformation. In terms of management strategies, nurse managers reported moving beyond ad hoc, experience‐driven approaches and increasingly adopting structured tools (e.g., attribution analysis/root cause analysis) to support problem tracing and process optimization. Santo et al. [41] noted that systematic management methods can strengthen early‐warning capacity while fostering structured thinking and forward‐looking governance among managers. Such tool‐supported practice may facilitate a shift from experiential decision‐making to strategic governance, thereby reinforcing a prevention‐oriented patient safety culture. In addition, participants described moving away from “one‐size‐fits‐all” management and instead tailoring communication and interventions to the involved nurse’s personality traits, career stage, and emotional state. This aligns with the “individualized care” approach described by Almost et al. [42], which suggests that adapting management responses to employees’ psychological characteristics and situational contexts can reduce tension and facilitate recovery. Role awareness also appeared to evolve through incident response and reflection. Nurse managers were no longer positioned solely as passive “fixers” who absorb responsibility after the fact; rather, they increasingly described themselves as proactive leaders who promote prevention systems and training mechanisms. Esquisábel‐Soteras [43] characterized this shift as an evolution “from frontline executor to system‐level governor.” Such role transformation may enhance nurse managers’ sense of professional value and strengthen their organizational belonging and commitment.

Such role development is shaped not only by the challenges inherent in patient safety incidents, but also by the organizational culture and institutional mechanisms that support managers after an event. Healthcare organizations should strengthen “postincident growth” mechanisms in routine practice by fostering a culture of reflection and embedding structured management training. In doing so, organizations can translate nurse managers’ experiential coping into institutionalized capability, building a learning‐ and growth‐oriented management workforce that provides a solid foundation for patient safety management.

Theme 4 captures system‐level recommendations proposed by nurse managers based on firsthand experience, aimed at strengthening individual–organizational collaboration and offering practical pathways for building an effective support system. Participants suggested that public, blame‐oriented experience‐sharing sessions rarely achieve meaningful learning and may instead inflict “secondary harm” on the involved nurses, intensifying shame, self‐blame, and anxiety—consistent with Li et al. [44]. The ERNST policy statement also emphasizes that improving patient safety should not remain at the level of individual blame but should instead promote the establishment of a just culture in healthcare settings through systemic change [45]. Accordingly, they recommended shifting toward learning‐oriented, nonpunitive debriefings, complemented by constructive feedback and positive reinforcement to minimize moral judgment. Participants further emphasized that psychological support should move beyond temporary consolation to an institutionalized, staged intervention program. They proposed tracking and addressing psychological distress at specific time points following an incident (e.g., 24 h, 7 days, and 60 days), which aligns with the second‐victim recovery pathway described by Scott et al. [6]. With regard to accountability, while the literature widely advocates nonpunitive reporting to protect involved staff, participants cautioned that overly diffuse accountability may inadvertently weaken responsibility education. They, therefore, called for clearer accountability arrangements, particularly for less‐experienced nurses, with responsibility aligned to competence—balancing protection of nurses’ rights with reinforcement of professional accountability. Although the Guidelines on Promoting High‐Quality Development of Public Hospitals [15] call for strengthened departmental collaboration and optimized management processes, our findings suggest persistent implementation gaps, including continuing barriers to interdepartmental cooperation. This echoes Zicko et al. [37], who noted that a robust safety culture requires cross‐departmental coconstruction yet is often insufficiently realized in practice. Therefore, establishing standardized, actionable collaboration and reporting procedures—with clearly defined roles and responsibilities across functional departments—may improve system‐level coordination and reduce the burden on unit‐level nurse managers during incident handling. Finally, participants noted that some hospitals still adopt a “one‐size‐fits‐all” approach to incident management, overlooking the need for differentiated responses according to incident type, scope, and severity of harm. They recommended implementing tiered management based on severity (e.g., minor incidents managed within the unit; major incidents escalated to the nursing department and quality‐control platform), together with structured patient follow‐up to monitor harm outcomes. This reflects a full‐cycle approach to managing patient safety incidents.

### 4.1. Strengths

This study focuses on nurse managers—a critical yet often overlooked group in the context of patient safety incidents—thereby addressing a limitation in prior research that has largely concentrated on frontline nurses. Using a phenomenological qualitative design and reporting in accordance with the COREQ checklist, the study enhances methodological transparency and credibility. Triangulation was applied across data collection and analysis to reduce potential bias and strengthen the trustworthiness of the findings. Importantly, the study translates participants’ experiences into actionable organizational recommendations, including strengthening psychological support, implementing tiered incident management, and improving interdepartmental collaboration, which may inform hospital policy‐making and system development.

### 4.2. Limitations

All participants were recruited from a single tertiary hospital in Nanjing, China, which may introduce regional bias and limit the generalizability of the findings. In addition, all 14 participants were female, resulting in a lack of gender diversity and the potential omission of gender‐related differences in stress coping and management styles. The data relied primarily on participants’ recollections, which may be subject to recall bias. In addition, only three participants were randomly selected for member checking; although their feedback was consistent and no new themes emerged, some divergent or minority perspectives may still have been missed. Finally, although the researchers maintained a stance of epoché throughout the study to minimize preconceived notions, the final findings inevitably represent the researchers’ interpretations of the participants’ narratives and may be influenced by their subjective understanding.

## 5. Conclusion

This study focused on the lived experiences of nursing managers during patient safety incidents, revealing their emotional distress, structural support deficiencies, and processes of role growth as “second victims.” It expands theoretical understanding of patient safety incidents from the perspective of the management tier. Healthcare institutions should urgently establish systematic support mechanisms, including psychological assessment and intervention, stress monitoring, positive reinforcement, and nonpunitive review systems. Furthermore, it is essential to institutionalize interdepartmental collaboration, fostering a safety culture characterized by shared responsibility and resource integration. Future research could adopt multicenter sampling to explore how nursing managers’ stress responses and support needs vary across different organizational cultures. Mixed‐method designs combining quantitative assessment with longitudinal approaches are also recommended to investigate the long‐term dynamics of psychological stress throughout the occurrence, response, and recovery phases, providing both theoretical foundations and practical pathways for strengthening patient safety systems.

## Funding

No funding was received for this manuscript.

## Conflicts of Interest

The authors declare no conflicts of interest.

## Data Availability

The data that support the findings of this study are available from the corresponding author upon reasonable request.
